# Structural and biophysical characterization of the secreted, β-helical adhesin EtpA of Enterotoxigenic *Escherichia coli*

**DOI:** 10.1371/journal.pone.0287100

**Published:** 2023-06-21

**Authors:** Clifford Manyo Ntui, James M. Fleckenstein, Wolf-Dieter Schubert

**Affiliations:** 1 Department of Biochemistry, Genetics and Microbiology, University of Pretoria, Pretoria, South Africa; 2 Department of Medicine, Division of Infectious Diseases Washington University in Saint Louis, School of Medicine, Saint Louis, Missouri, United States of Ameirca; 3 Infectious Disease Service Saint Louis VA Health Care System, Saint Louis, Missouri, United States of Ameirca; University of Queensland, AUSTRALIA

## Abstract

Enterotoxigenic *Escherichia coli* (ETEC) is a diarrhoeal pathogen associated with high morbidity and mortality especially among young children in developing countries. At present, there is no vaccine for ETEC. One candidate vaccine antigen, EtpA, is a conserved secreted adhesin that binds to the tips of flagellae to bridge ETEC to host intestinal glycans. EtpA is exported through a Gram-negative, two-partner secretion system (TPSS, type Vb) comprised of the secreted EtpA passenger (TpsA) protein and EtpB (TpsB) transporter that is integrated into the outer bacterial membrane. TpsA proteins share a conserved, N-terminal TPS domain followed by an extensive C-terminal domain with divergent sequence repeats. Two soluble, N-terminal constructs of EtpA were prepared and analysed respectively including residues 67 to 447 (EtpA^67-447^) and 1 to 606 (EtpA^1-606^). The crystal structure of EtpA^67-447^ solved at 1.76 Å resolution revealed a right-handed parallel β-helix with two extra-helical hairpins and an N-terminal β-strand cap. Analyses by circular dichroism spectroscopy confirmed the β-helical fold and indicated high resistance to chemical and thermal denaturation as well as rapid refolding. A theoretical AlphaFold model of full-length EtpA largely concurs with the crystal structure adding an extended β-helical C-terminal domain after an interdomain kink. We propose that robust folding of the TPS domain upon secretion provides a template to extend the N-terminal β-helix into the C-terminal domains of TpsA proteins.

## Introduction

Enterotoxigenic *Escherichia coli* (ETEC) is a leading cause of infectious diarrhoea in young children of developing countries, leading to acute mortality [1], as well as long-term sequelae including enteropathic changes to the small intestine that lead to nutrient malabsorption and growth impairment [2, 3]. Acute diarrhoeal symptoms are elicited by ETEC by the effective delivery of its heat-stable and heat-labile enterotoxins to intestinal epithelial cells. ETEC first colonises the host small intestine by attaching through fimbrial as well as non-fimbrial adhesins [4]. One such adhesin, EtpA [5], is a 177 kDa or 1767 residues, secreted protein that bridges the tips of ETEC flagella to host epithelial [6] glycans including human A blood group to promote bacterial adhesion, intestinal colonization and toxin delivery [7, 8].

EtpA is secreted as part of a Gram-negative, type Vb or two-partner secretion system (TPSS) found in human pathogens such as *Haemophilus influenza* [9], *Bordetella pertussis* [10] and ETEC [11, 12]. A TPS typically comprises a secreted exoprotein (TpsA) of 100 kDa or more and a ∼60-kDa transporter protein (TpsB) [13]. Both TpsA and TpsB carry N-terminal signal sequences for Sec secretion across the inner membrane [14, 15]. TpsB proteins like other members of the Omp85 superfamily are inserted into the outer membrane of Gram-negative bacteria, and consist of a C-terminal integral membrane β-barrel and an N-terminal “polypeptide transporter-associated” (POTRA) domain [16]. Outer membrane TspB proteins recognise the N-terminal TPS domain of their cognate TpsA to initiate exoprotein translocation across the outer membrane [17] with simultaneous folding nucleated by the N-terminus [18]. All structurally resolved TpsA TPS domains form right-handed β-helices with occasional α-helical additions. They include FHA30 of *B*. *pertussis*, HpmA265 of *Proteus mirabilis*, as well as Hmw1-PP and HxuA of *H*. *influenza* [19–22]. The TPS is typically followed by long stretches of imperfect repeats (*e*.*g*., four 228-residue repeats in EtpA). The study of TpsA proteins has been hampered by their large size and the need for cognate TpsBs. HxuA, the smallest TpsA at 96 kDa is the only protein for which the full-length structure is known [22]. TpsA proteins form two subfamilies by sequence but functionally cluster into cyto-/hemolysins, adhesins (including EtpA), proteases, iron acquisition, and contact-dependent growth inhibition proteins [13].

Here we report the production of two overlapping N-terminal EtpA fragments, EtpA^1-606^ and EtpA^67-447^, both containing the TPS domain (S1 Fig). By gel filtration chromatography, dynamic light scattering and circular dichroism spectroscopy we demonstrate that secreted EtpA^1-606^ is thermostable and refolds reversibly when unfolded in urea. The crystal structure of EtpA^67-447^ reveals a β-helical structure while AlphaFold modelling of TpsA proteins reveals a common feature of continuous, yet repetitive β-helical structures.

## Methods

### EtpA constructs

Two N-terminal EtpA fragments were constructed and purified, EtpA^67-447^ and EtpA^1-606^, respectively consisting of residues 67 to 447, and 1 to 606. EtpA^1-606^ was produced from a pBAD*/*Myc*-etpBA-*His plasmid (James M. Fleckenstein, Washington University, St Louis, MO, USA) which encodes the EtpB transporter protein and residues 1–606 of EtpA plus a C-terminal His_6_ tag. The signal peptide residues 1–66 are predicted to be removed during Sec secretion [23]. The DNA fragment encoding EtpA^67-447^ was PCR-amplified (S1 Table) from the existing *etpBA* plasmid [5] and directionally cloned into the pGEX-6P-2 expression vector (Cytiva, Marlborough, MA, USA) *via BamH1* and *Not1* restriction sites.

### Protein production and purification

Following transformation of *Escherichia coli* BL21 cells (Life Technologies, CA, USA) with the pGEX-6P-2-*etpA*^67-447^ plasmid, transformed cells were grown at 37°C in LB medium with 100 mg/L ampicillin to an OD_600_ of 0.8 then induced with 0.2 mM isopropyl β-D-1-thiogalactopyranoside (IPTG) followed by shaking at 22°C for 18 h. The cell pellet was resuspended in PBS buffer, lysed by sonication, and centrifuged at 37 000 x *g* (Sorvall Lynx 6000, ThermoFisher Scientific) for 1 h at 4°C. The soluble supernatant was mixed with glutathione sepharose (GS) resin (Cytiva) and incubated with agitation for 1 h at 4°C for GST binding to GS beads. Unbound protein was removed by extensive washing with PBS in a gravity flow column. Target EtpA proteins were released by 0.1 mg 3C protease (University of Pretoria) for 24 h at 4°C with agitation, eluted with PBS, dialysed against 20 mM Tris pH 8.0, 20 mM NaCl and further purified by ion-exchange chromatography using a HiTrap Q HP column (Cytiva) and a linear gradient of 5 to 1000 mM NaCl in 20 mM Tris pH 8.0. Pooled EtpA fractions were further analysed by size exclusion chromatography (SEC) in 10 mM MES pH 5.5, 25 mM NaCl and 5% (v/v) glycerol using an Enrich SEC 650 (10 x 300) column (Bio-Rad) on an Äkta pure system (Cytiva) and concentrated in Amicon Ultra-15 filters (Merck, Germany) to 18 mg/mL.

For EtpA^1-606^, *Escherichia coli* TOP10 cells (Life Technologies) were transformed by heat shock using the pBAD-*etpBA* plasmid. Cells were grown at 37°C in LB medium with 100 mg/L ampicillin. Production of EtpA^1-606^-His_6_ was induced at OD_600_ 0.5 with 0.002% (w/v) arabinose. Cultures were shaken at 22°C for 18 h. The N-terminal EtpA-His_6_ protein was recouped by centrifuging the bacterial cell culture at 10 000 x *g* (Sorvall Lynx 6000) for 20 min at 4°C, decanting the supernatant and adding 0.5 mM PMSF (Merck, Germany). Clean Ni-NTA beads were added and the mix incubated with agitation for 1 h at 4°C. Unbound protein was eluted with 20 mM Tris pH 7.9, 200 mM NaCl in a gravity flow column. Target EtpA^1-606^ was eluted with 20 mM Tris pH 7.9, 500 mM NaCl, 200 mM imidazole. The protein was concentrated in Amicon Ultra-15 filters.

### Size determination and oligomeric state

The molecular size and the oligomeric state of EtpA^1-606^ were assessed using SDS-PAGE, native-PAGE, size exclusion chromatography (SEC) and dynamic light scattering (DLS). For SDS-PAGE, loading dye with SDS was mixed with protein samples, heated for 5 min at 95°C and analysed by SDS-PAGE. For the native-PAGE, loading dye without SDS was mixed with protein samples and analysed on a native-PAGE without heating. For SEC, 4 mg EtpA^1-606^ was analysed by Enrich SEC 650 (10 x 300) column (Bio-Rad) on an Äkta pure system (Cytiva). The column was pre-equilibrated with 20 mM Tris pH 7.4 and 150 mM NaCl at 0.2 mL/min. The size distribution of EtpA^1-606^ was analysed by dynamic light scattering (DLS) in a Zetasizer 7.13 (Malvern Panalytical, UK) at 4°C using 1 mg/mL protein in 20 mM Tris pH 7.5, 150 mM NaCl.

### Unfolding and refolding studies

Proteins were thermally unfolded and refolded in buffer A (10 mM phosphate buffer pH 7.4, 150 mM NaCl). Circular dichroism (CD) spectra were recorded in a 1-mm cuvette in a Chirascan spectrophotometer (Applied Photophysics, UK) for 180 to 280 nm at 0.25 s/nm repeated fourfold. For thermal unfolding, 1 mg/mL EtpA^1-606^ was heated from 20 to 100°C at 10°C intervals in a temperature-controlled compartment. The observed ellipticity at 222 nm for each temperature was plotted against temperature to determine the transition mid-temperature (T_m_) of EtpA^1-606^. Urea-induced protein unfolding was studied in buffer A with 0 to 9 M urea. The absorbance of EtpA^1-606^ both with and without urea were recorded between 180 and 280 nm against respective blanks at 20°C after 24 h. Samples were prepared by mixing 250 μL 8 mg/mL EtpA^1-606^ with an appropriate volume of urea and adjusting to a final volume of 2 mL with buffer A. To assess the reversibility of the unfolding process, 1 mL of the unfolded protein incubated in the respective urea concentration for 24 h was diluted tenfold with buffer A at 20°C and incubated for 24 h with gentle agitation. The urea was removed by extensive dialysis against buffer A for 24 h at 20°C and by repeated dilution and concentration in a 50 kDa Amicon Ultra-15 concentrator. The final protein concentration was 0.8 mg/mL. The absorbance of the refolded samples was recorded at 180 to 280 nm at 20°C using buffer A as blank.

### Crystallization, data collection and processing, structure determination and refinement

Lead crystallization conditions for EtpA^67-447^ at 12°C were obtained by hanging-drop vapour-diffusion experiments using the Procomplex screen kit (Qiagen). Optimized crystallization conditions combined 2 μL EtpA^67-447^ (15 mg/mL in 10 mM MES pH 5.5, 25 mM NaCl, and 5% (v/v) glycerol) with 2 μL reservoir solution (0.1 M Na_2_ citrate pH 5.5, 20% (w/v) PEG 4000, 20% (v/v) isopropanol). Crystals for X-ray diffraction were cryocooled in liquid nitrogen.

### Diffraction data were recorded remotely on an Eiger2 XE 16M detector on beamline i04, DIAMOND Light Source (Oxfordshire, UK). Images were auto processed, scaled and merged using the Xia2 XDS program suite [24]. For structure determination see the Results section

### AlphaFold models

Full-length theoretical models of EtpA (Genbank Accession: AAX13509.2) and the four TpsA members FHA (*B*. *pertussis*) (CPN83729.1) [25], HpmA (*P*. *mirabilis*) (SUC39485.1) [26], HMW1(Q48031) [27, 28] and HxuA (*H*. *influenza*) (Protein Data Bank: 4RM6) were generated using AlphaFold [29].

## Results

### Protein production, purification, size and oligomerisation

This study involved two overlapping N-terminal and TPS domain encompassing EtpA fragments: EtpA^1-606^ and EtpA^67-447^. Unfolding, refolding, SEC and DLS studies where limited to the original, longer and secreted EtpA^1-606^ fragment. The intracellularly produced EtpA^67-447^ was only generated after crystallization experiments with EtpA^1-606^ failed. The larger size of EtpA^1-606^ and the fact that it is secreted by the native Type 5b secretion system could mean that it more fully reflects the properties of full-length EtpA. The difference in oligomerization behaviour of EtpA^1-606^ compared to monomeric EtpA^67-447^ (see below) is, though, not entirely clear.

**Fig 1:**
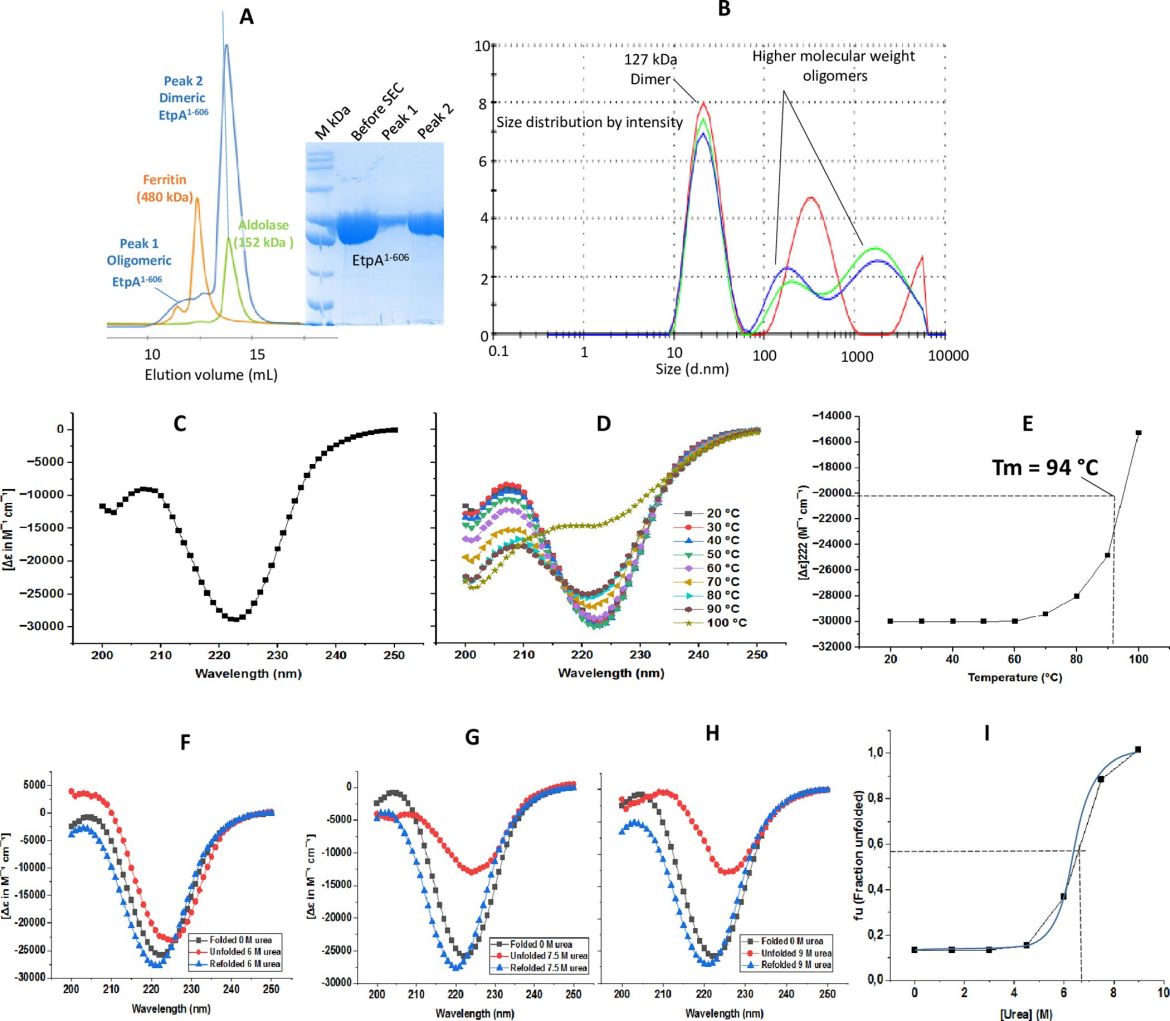
Purification and biophysical characterisation of EtpA^1-606^. **(A)** Size exclusion chromatography of EtpA^1-606^ with aldolase (152 kDa, green) and ferritin (480 kDa, orange) as standards. The retention volume indicates stable EtpA^1-606^ dimers. Insert: SDS-PAGE of EtpA^1-606^ peak fractions before and after SEC. **(B)** Dynamic light scattering profiles confirm EtpA^1-606^ dimers plus higher molecular weight oligomers. **(C)** A broad minimum around 222 nm in the CD spectrum for EtpA^1-606^ indicates a β-helical fold. **(D)** Thermal unfolding data between 20 and 100°C. **E)** Ellipticity at 222 nm plotted against temperature yields a T_m_ of ~94°C. **(F-H)** CD spectra for untreated (0 M urea, black squares), urea treated (6 to 9 M urea, red spheres) and renatured samples (blue triangle). **(I)** Unfolding curve for EtpA^1-606^ obtained after unfolding the protein.

EtpA^67-447^, purified by glutathione sepharose (GS) affinity, ion exchange and size exlusion chromatography yielded a single band on SDS-PAGE with an expected size of ~38 kDa (S2 Fig). EtpA^1-606^ was purified by Ni-NTA affinity and size exclusion chromatography (SEC). SEC revealed multiple peaks with retention volumes 11.6, 12.6 and 13.8 mL (Fig 1A) all due to EtpA^1-606^ (insert). SEC column calibration indicated molecular masses of ~504, 320 and 160 kDa or octameric, tetrameric and dominant dimeric EtpA^1-606^, respectively. Dynamic light scattering (DLS) confirmed the EtpA^1-606^ dimers alongside high molecular weight (HMW) aggregates (Fig 1B).

### Temperature-induced unfolding of EtpA^1-606^

The thermal stability of EtpA^1-606^ was tracked using circular dichroism (CD) spectroscopy. At 20°C, a single broad minimum at ~222 nm (Fig 1C) was observed, typical for β-helical structures [30]. Spectra up to 100°C demonstrate that EtpA^1-606^ remained folded to ~90°C before rapidly unfolding (Fig 1D). A plot of ellipticities at 222 nm against temperature (Fig 1E) yielded a T_m_ of 94°C for EtpA^1-606^ indicating a highly thermostable protein.

### Urea dependent unfolding and refolding of EtpA^1-606^

The chemical stability of EtpA^1-606^ was assessed using urea concentrations up to 9 M. For each urea concentration, CD spectra for untreated (fully folded), urea-treated (partly unfolded) and dialysed (refolded) samples were recorded (Fig 1F to 1H and S3 Fig). EtpA^1-606^ remained largely intact up to 4.5 M urea (S2 Fig) with increasing degrees of denaturation for 6 to 9 M urea (Fig 1F–1H). Plotting the fraction of unfolded protein (fu) against the urea concentration indicates a critical urea concentration of ~7 M (Fig 1I). Interestingly, urea-induced unfolding of EtpA^1-606^ is mostly reversible up to 9 M urea, demonstrated by the blue curves returning to the original curve after dialysis (Fig 1F–1H and S3 Fig).

### Structure determination of EtpA^67-447^

**Fig 2:**
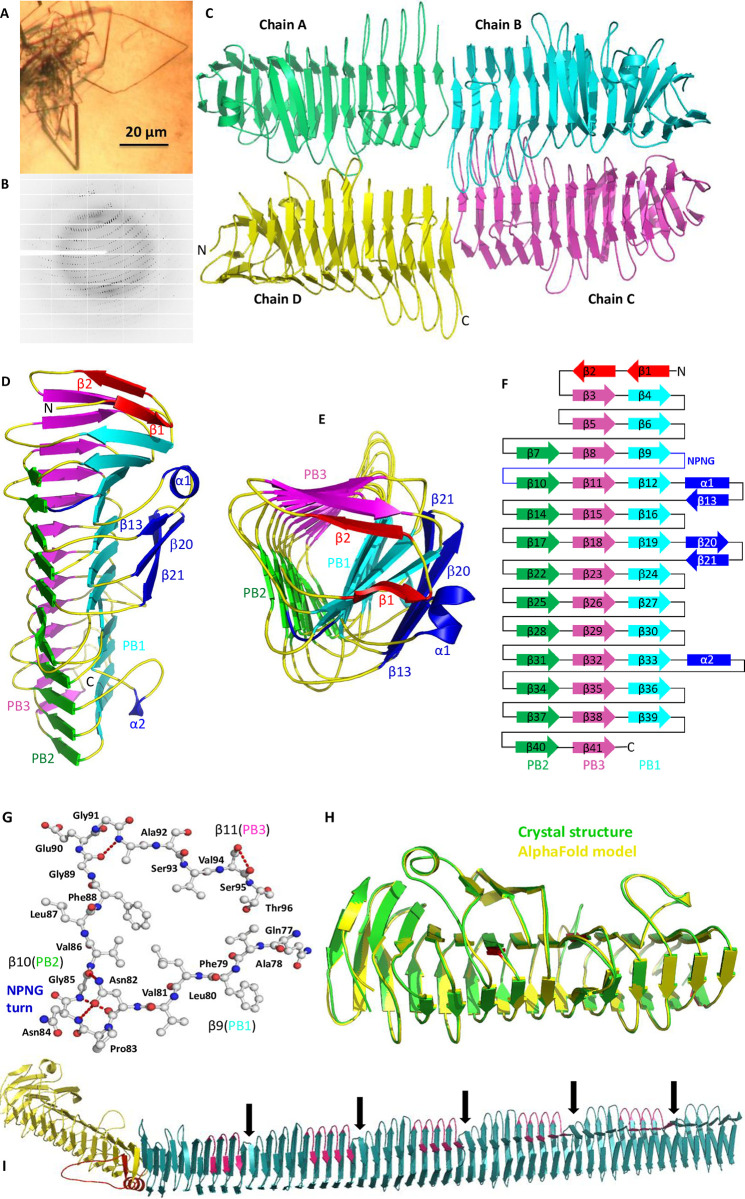
Structure of EtpA. **(A)** EtpA^67-447^ crystals, **(B)** X-ray diffraction pattern, **(C)** Four EtpA^67-447^ molecules occupy the asymmetric unit of the crystal structure. **(D)** Side-view of EtpA^67-447^ with β-sheets PB1, PB2 and PB3 shown in orange, green and magenta; extra-helical motifs α1/β13/β20/β21 and conserved NPNG motif in blue. **(E)** N-terminal view emphasizing terminal β-strands β1 and β2 shielding the hydrophobic core. **(F)** EtpA^67-447^ topology. **(G)** Ball-and-stick view of β-helix turn 4 with the conserved NPNG motif in loop β9-β10. Red, dotted lines mark turn-stabilising hydrogen bonds. **(H)** Structural alignment of the experimental TPS domain crystal structure in green and the AlphaFold model in yellow. Structural differences in β-strands are marked in red. **(I)** AlphaFold model of full-length EtpA. The N-terminal domain in yellow harbours the TPS domain. The repetitive C-terminal domain is in dark cyan. An intervening α-helix, linked to the interdomain kink, and the associated loop are highlighted in red. Conserved STSGNAINL motifs associated with C-terminal repeats are shown in magenta. Indentations following major repeats are marked by black arrows. Structures were visualized on UCSF Chimera and images were prepared in Pymol.

EtpA^67-447^ crystal platelets grew within two weeks at 12°C and diffracted X-rays to 1.8 Å at beamline i04 (DIAMOND Light Source, UK) (Fig 2A, 2B and Table 1). Diffraction data were processed by the Xia2 XDS pipeline [24]. The crystal structure of EtpA^67-447^ was determined by molecular replacement in PHENIX Phaser-MR [31] using the crystal structure of HMW1A-PP (PDB code 20DL) with 34% sequence identity as search model. The model was re-built and refined by PHENIX Autobuild followed by manual adjustment in WinCoot [32] and further refinement in PHENIX refine. Data collection and refinement statistics are summarised in Table 1. The molecular replacement was repeated with the AlphaFold EtpA^67-447^ model with equivalent results.

**Table 1:** Data collection and refinement statistics for EtpA^67-447^.

Data collection statistics	
Wavelength (Å)	0.9795
Resolution range (Å)*	61.95–1.76 (1.82–1.76)
Space group	P1
Unit cell (a, b, c, α, β, γ) (Å, °)	37.7, 64.1, 123.9, 91.6, 90.9, 90.0
Images collected	3600
Total reflections*	382586 (35589)
Unique reflections*	111780 (10743)
Multiplicity*	3.4 (3.3)
Completeness (%) *	96.8 (93.0)
Mean I/sigma(I)*	8.4 (0.6)
Wilson B-factor	24.1
R-merge*	0.1 (1.4)
R-meas*	0.12 (1.6)
**Refinement statistics**	
Reflections used for R-free*	111589 (10742)
R-work*	0.20 (0.34)
R-free*	0.24 (0.37)
No. of non-hydrogen atoms	12252
No. of water molecules	702
No. of amino acid residues	1533
RMS (bonds)(Å)	0.009
RMS (angles) (°)	0.80
Ramachandran favoured, additionally allowed, outliers (%)	96.50, 3.40, 0.00
Average B-factor for protein (Å^2^)	32.3
Average B-factor for solvent (Å^2^)	39.7

*Values in parentheses are for the outermost resolution shell.

The asymmetric unit of the EtpA^67-447^ crystals contained four symmetrically independent but equivalent monomers (Fig 2C). Each monomer forms a triangular, right-handed β-helix (Fig 2D) of 13 turns (or rungs), where each turn consists of two or, more commonly, three roughly coplanar β-strands. Strands from adjacent turns align to create the parallel β-sheets PB1, PB2 and PB3 with 13, 11 and 14 β-strands, respectively. Three β-strands of EtpA^67-447^, β13, β20 and β21, create a small extra-helical β-sheet (blue in Fig 2D–2F). β-Strands β1 to β8 create a tapered N-terminal end of the β-helix slightly tilted from the main β-helical axis. While β-strands β1 and β2 (red) form part of PB1 and PB2, they align antiparallel to the otherwise parallel β-sheets and serve to cap the β-helix.

All 38 β-helical β-turns consist of four residues mostly including one or two glycine residues and are stabilized by hydrogen bonds between glycine main chain atoms and serine side chains oriented towards the β-helix interior. The loop connecting β-strands β9 and β10 is a type 1 β-turn and harbours a conserved NPNG motif critical for TpsA protein folding [33]. The loop is stabilized by hydrogen bonds between the loop main chain and side chains of N82, N84, G85, G89, G91 and S95 (Fig 2G). PB1 and PB2 β-strands are three to seven residues in length, with those of PB2 decreasing in length towards the C-terminus. The interior of the β-helix is dominated by hydrophobic, aliphatic residues, especially valine. N-terminally, β-strands β1 and β2 linked by a hairpin turn shield the hydrophobic core of the β-helix.

Two loops connecting physically adjoining β-strands β12-β14 and β19-β22, are extended to create an extra helical domain consisting of α-helix α1 and β-strands β13, and β20 and β21 (Fig 2D), respectively. The extra-β-helical secondary structure elements all pack onto the outer face of β-sheet PB1 broadly alligned (anti-) parallel to its β-strands. Helix α1 is positioned by hydrogen bonds between Asn108 (α1) and Arg59 (β6 in PB1). Similar hydrogen bonds anchor β-hairpin strands β20 (Arg169 and Thr171) and β21 (Gln183 and Thr185) to β13 (Lys161). A second extra-helical motif involves α-helix α2 within loop β33-β34 (Fig 2F).

### Modelled structure of full-length EtpA

Structural analysis of full-length EtpA was attempted by removing three of four C-terminal repeats of the encoding gene construct. However, intracellular production failed, yielding insoluble protein only. A structural model of full-length EtpA was instead generated by AlphaFold [29]. The TPS domain represented by the EtpA^67-447^ crystal structure closely matches the corresponding part of the AlphaFold model (Fig 2H) despite minor differences in β-strands lengths (red in Fig 2H). The AlphaFold model of EtpA extends the β-helical structure of the TPS domain to the entire C-terminal region of the protein (cyan in Fig 2I), though the two regions are separated by an extended loop bearing an α-helix that is partly wedged between the N- and C-terminal β-helical domains (red in Fig 2I), creating a pronounced interdomain kink. The EtpA C-terminal section consists of four large 228 residue repeats composed of nine β-helical turns each, four of which contain a conserved STSGNAINL motif (magenta in Fig 2I). Larger repeats are terminated by a constriction of the β-helix (arrows). Extra-helical domains observed for the EtpA N-terminal region are absent in the C-terminus.

## Discussion

### EtpA N-terminal domain and related structures

The N-terminal fragment EtpA^1-606^ proved highly soluble and stable with a T_m_ of 94°C and C_m_ of -21000 M¯^1^ cm¯^1^. Interestingly EtpA^1-606^ denaturation was fully reversible, implying a clear folding path and supporting its inherent stability. A stable β-helical fold would seem particularly suited to a secreted protein having to survive and achieve its role in infection in an unpredictable environment. Stability or folding analyses for other TpsA proteins are currently lacking. However, extensive studies of structurally related pertactin, an autotransporter (type 5a secretion) effector domain from whooping cough causing *Bordetella pertussis* revealed a similarly stable C-terminal domain that functions as a template for the efficient β-helix formation [34, 35]. (Autotransporter effector secretion starts C-terminally–compared to N-terminal secretion for most other systems including EtpA.) Similar to pertactin, the stable EtpA^1-606^ fold appears critical in achieving efficient secretion and vectorial folding of full-length EtpA. β-Helical proteins beyond TpsAs include meso- and thermo-stable pectate lyases [36] or heat and chaotrope resistant “gene product 5” (Gp5), a spike-shaped trimeric bacteriophage T4 protein [37]. Like EtpA, Gp5 has a well-defined repeat, refolds spontaneously, and forms oligomers [37].

**Fig 3:**
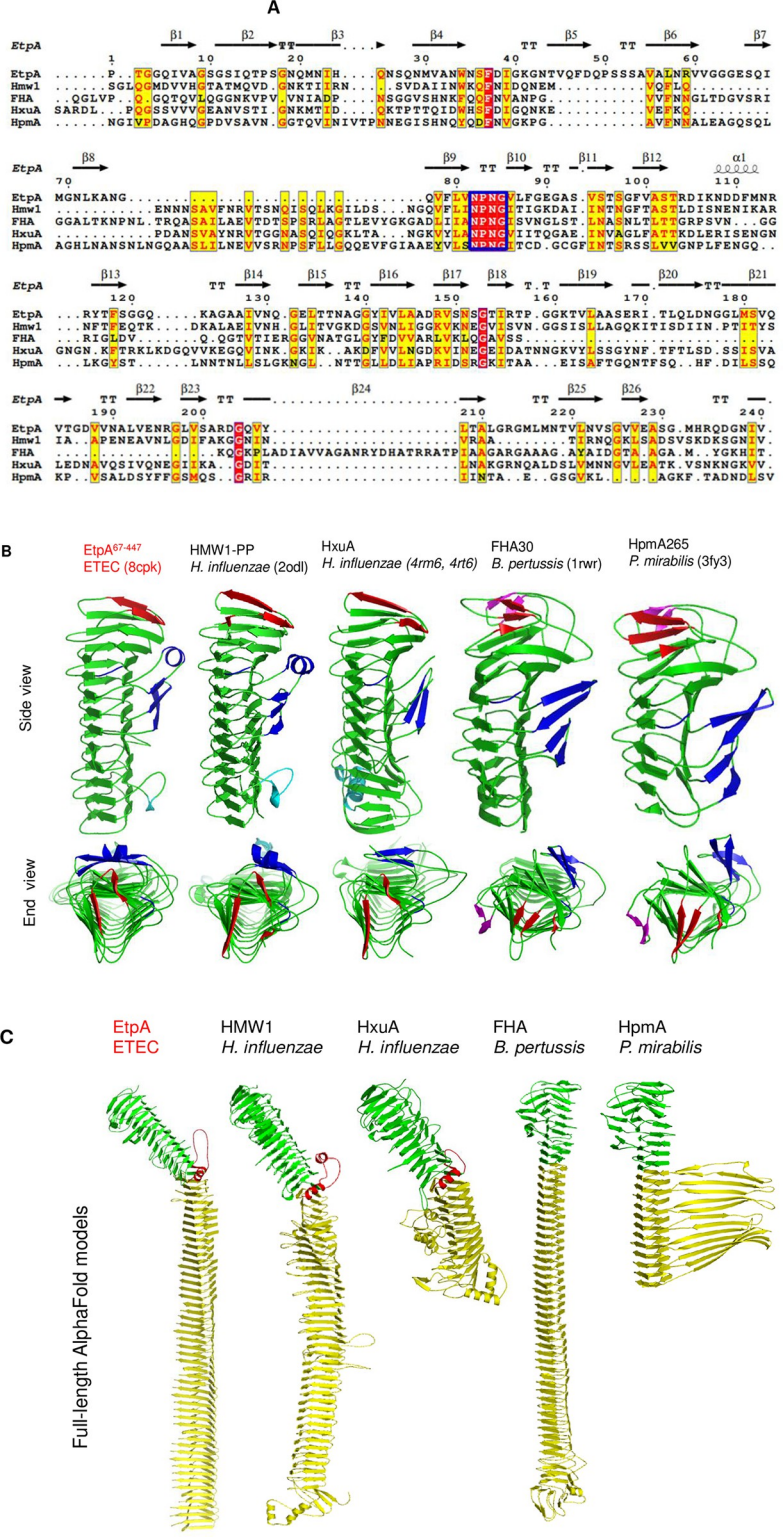
Comparison of selected TpsAs. **(A)** A partial structure-based sequence alignment of the TPS domain of EtpA^67-447^, HxuA301, HMW1-PP, HpmA265 and Fha30. Conserved and partly conserved amino acids are shown as white letters on a red background and red on yellow. A blue box marks the conserved NPNG motif. **(B)** TpsA TPS domain crystal structures. Above: Lateral views, below: N-terminal views. The β strands of the N-terminal cap are shown in red, the conserved NPNG motifs and the extra-helical domains in blue, partly-conserved NPNL motifs in magenta, additional extra-helical elements in cyan. **(C)** AlphaFold models of full length proteins. TPS containing N-terminal and C-terminal domains are in green and yellow, respectively, interdomain α-helices and adjoining loops in red.

The crystal structure of EtpA^67-447^ is the fifth TpsA TPS crystal structure overall. The four previous TpsA TPS structures include those of high molecular weight adhesin HMW1A (HMW1-PP) [18] and hemopexin-binding protein, HxuA, from *H*. *influenza* [21, 22], of filamentous hemagglutinin adhesion FHA from *B*. *pertussis* (FHA30) [19], and of hemolysin HpmA from *P*. *mirabilis* (HpmA265) [20]. Despite low sequence identities of EtpA^67-447^ with the other TPS domains (34% for HMW1-PP, 35% for HxuA, 27% for FHA30, and 23% for HpmA265, Fig 3A), the domains all form analogous right-handed β-helices (Fig 3B) with tightly packed, hydrophobic cores and characteristic aromatic clusters in the first helical turn (W34, F47, F79, F88 and F99 in EtpA). N-terminal caps that shield the hydrophobic cores differ in the number and arrangement of β-strands (Fig 3B). In EtpA^67-447^ and HxuA two β-strands protect the core, while in Hmw1-PP, FHA30 and HpmA265 three β-strands are involved. These differences may reflect distinct co-evolution with their TpsB outer membrane transporters [13]. TPS domains also share a conserved NPNG motif, the first asparagine of which is essential to maintain secretion rates of FHA and ShalA [33, 38]. A related NPNL motif observed in FHA30 and HpmA265 is, however, absent in EtpA^67-447^, HMW1-PP and HxuA. TpsAs furthermore share similarly positioned extra-helical domains (blue in Fig 3B). Though the number and arrangement of secondary structural elements and their insertion points within the β-helix vary considerably, their consistent placement alongside β-sheet PB1 would nevertheless imply a common role including possible homotypic interactions for biofilm formation [39]. Extra-helical motifs also appear to provide evolutionary hotspots to generate additional binding sites especially as the structural rigidity of the β-helix itself limits the evolution of additional functions. The bridging interaction of EtpA with flagellin molecules and host glycans to promote adhesion and toxin delivery presumably involves unique, possibly repeating motifs within this large exoprotein. Further functional characterisation of TpsA proteins will be required to reveal the role of individual secondary structural elements in recognition, secretion, folding and additional functions.

### Full-length structural models of TpsA proteins

The large size, poor solubility and the requirement for specific TpsB transporters have historically complicated the structural analysis of full-length TpsAs such that only HxuA, the smallest member of the family, ever yielded a full-length crystal structure [22]. Correspondingly, the roles of the C-terminal domains are currently not well understood. In HxuA this domain binds hemopexin via the extra-helical motifs [22]. AlphaFold and RosettaFold modelling servers [29, 40] now generate theoretical structural models of full-length TpsAs (Fig 3C), expanding the previously available experimental TPS domain structures. Apart from EtpA, we generated structural models for HMW1, HxuA, FHA and HpmA. In all these proteins, the β-helical fold observed in their TPS domains (Fig 3B) is seen to extend into the C-terminal domains (Fig 3C). TpsA C-terminal β-helices, however, differ appreciably both in length and repeat-pattern. For HpmA, theoretical model predict a large, extended β-sheet extending laterally away from the β-helix (Fig 3C). While the fold is supported by the RosettaFold modelling server, its unorthodox structure would need to be confirmed experimentally. EtpA, HMW1A and HxuA all share interdomain α-helices as part of an extended loop(red in Fig 3C) that create a kink in the β-helix between N- and C-terminal domains. The kink is most pronounced in EtpA but less so in HMW1 and HxuA (Fig 3C). HpmA and FHA lack this α-helix, resulting in essentially linear assemblies (Fig 3C). In EtpA and HMW1 the β-helix constricts noticeably at the start of the C-terminal domain while the β-helical diameter continues largely unchanged in HxuA, HpmA and FHA, creating a single, continuous and linear β-helix. Apart from the interdomain kink and the constriction of the β-helix, the EtpA C-terminal domain is most similar to HMW1A and FHA in particular with respect to the length and linearity of the C-terminal domain. Functionally, EtpA shares adherence and agglutination properties with HMW1 and FHA [7, 41, 42].

The shape of proteins created from fused repeats may range from linear to circular, helical or twisted based on repeated contributions from each repeat unit [43]. Bends and twists are caused by gradual shifts in repeat proteins through multiple offsets leading to circular or helical rotation around the helix axis. By contrast, kinks create a singlular and localized offset of the helical axis. While bends and twists generally have little effect on protein stability, kinks often do [44] perhaps, in part, explaining the lack of crystal structures of β-helical effector proteins. EtpA and HMW1 with discernable kinks may thus prove less stable than their more linear counterparts. Antigen 43a, a β-helical adhesin from *E*. *coli* involved with cell aggregation and biofilm formation is also kinked [45], implying that the kink of EtpA may be of functional importance [45].

Apart from subtype Vb secreted TpsA effectors, β-helical structures are also typical for passenger domains of the related autotransporters (ATs) or type Va secretion systems such as pertactin, antigens 43a (4KH3) and 43b (7KOB) as well as SepA (5J44) [45, 46]. β-Helical proteins beyond Type 5 effectors include tail spike protein of *E*. *coli* bacteriophage HK620 (2VJI) [47] and the AFP antifreeze protein [48]. A common feature of these β-helical proteins is their physical stability in a challenging extracellular environment. The thermodynamics of β-helix formation appears to aid secretion by providing a ratchet mechanism helping to move the protein through the pore in a single direction and offsetting a lack of accessible energy outside the outer membrane [49].

TpsA proteins typically contain complex sequence repeats with those in EtpA being among the longest. Repeat domains, including those of TpsAs, presumably evolve by internal gene duplications and recombination processes [43] creating longer proteins with larger surface areas for interaction with other proteins or surfaces. A large variety of proteins repeats are observed especially with sizes above 500 amino acids [50], critically contributing to their function [51]. Repeats vary from single amino acids, to short repeats of 20 to 40 amino acids, and larger repeats of more than 100 amino acids [51]. Structurally, repeats can include distinct domains of defined structure and function linked by unstructured loops [52] or directly fused repeating units as observed in EtpA. Protein functions linked to repeats include protein-protein interactions, nucleotide-binding, signal transduction, antiviral response and virulence [43]. Some repeat-rich proteins including EtpA are antigenic [53, 54]. Notably, repeat regions are common features of microbial lectins that target a variety of host cell glycan structures [55–57]. In the case of EtpA, its N-terminal domain is sufficient for it to bind to flagellin on the bacterial surface [6], while its C-terminal repeats presumably interact with human blood group A glycans [7].

An early hypothesis for TpsA translocation and folding suggested that TPS domains remain bound to POTRA domains while the remaining protein is translocated and folded on the cell surface [58]. Alternatively, TPS domains are thought to initiate folding upon secretion [13, 18]. This view is supported by the stable TPS fold, the efficient secretion of truncated N-terminal domains of TpsA proteins, TPS domain-initiated TpsA folding *in vitro* [20, 59], and the accessibility of N-termini of stalled FHA constructs at the cell surface [60, 61]. In this way the TPS domain can also serve as a folding template for the C-terminal domain.

In summary, we used CD spectroscopy to show that the EtpA N-terminal TPS domain forms thermostable and urea-stable folds that efficiently refold after denaturation. We further used X-ray crystallography and structure modelling by AlphaFold to demonstrate that the β-helical structure of the EtpA and related N-terminal domains provide a possible template upon which the C-terminal domain can efficiently fold. Accumulating structural data on EtpA and related virulence factors provide an increasingly clearer understanding of this important family of proteins. These concepts could inform the design of novel microproteins for vaccine development. While β-helical fragments remain challenging to produce, their inherent stability once formed could offer highly attractive vaccine candidates against a range of pathogens. While N- and C-terminal capping domains would inevitably be required to stabilize such mini-proteins the combination with an N-terminal fragment of a readily folding protein could help to successfully nucleate the β-helical folding process.

## Supporting information

S1 Table Plasmids and primers used. (TIF)

S1 Fig Schematic representation of full-length EtpA protein. The signal peptide (SP) for localization and processing and the TPS domain for recognition by the transport partner are indicated. The four consecutive repeats (R1, R2, R3 and R4) and the C-terminal tail are also indicated. The numbers represent the start and end of each fragment. The two N-terminal fragments; EtpA^1-606^ and EtpA^67-447^ are shown.
(TIF)

S2 Fig Purification of EtpA^67-447^. **(A)** Single-peak ion exchange chromatography profile, **(B)** Single-peak size exclusion chromatography profile of EtpA^67-447^ and a single band on SDS-PAGE (insert) matching the 38 kDa size of monomeric EtpA^67-447^.
(TIF)

S3 Fig Urea dependent un- and refolding of EtpA^1-606^. **(A-C)** CD spectra for untreated (0 M urea, black squares), urea treated (1.5 to 4.5 M urea, red spheres) and renatured samples (blue triangle).
(TIF)

S1 Raw images (PDF)
