# Supplementary figures and images for: Structural and biophysical characterization of the secreted, β-helical adhesin EtpA of Enterotoxigenic Escherichia coli
Source: PLoS One. 2023 Jun 21;18(6):e0287100. doi: 10.1371/journal.pone.0287100 (PMC10284417; doi:10.1371/journal.pone.0287100)

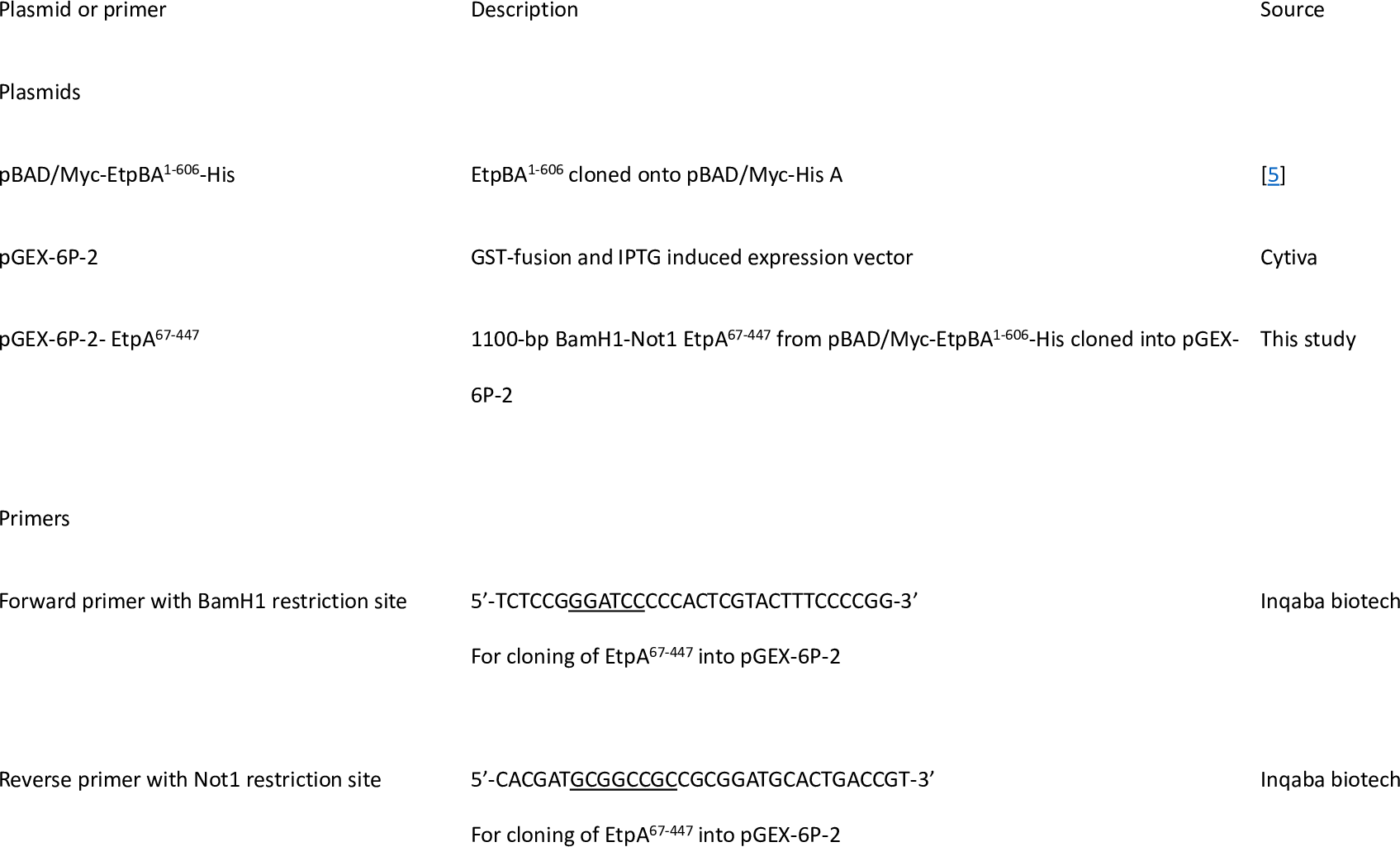

Supplement: S1 Table — (TIF) [file pone.0287100.s001.tif]

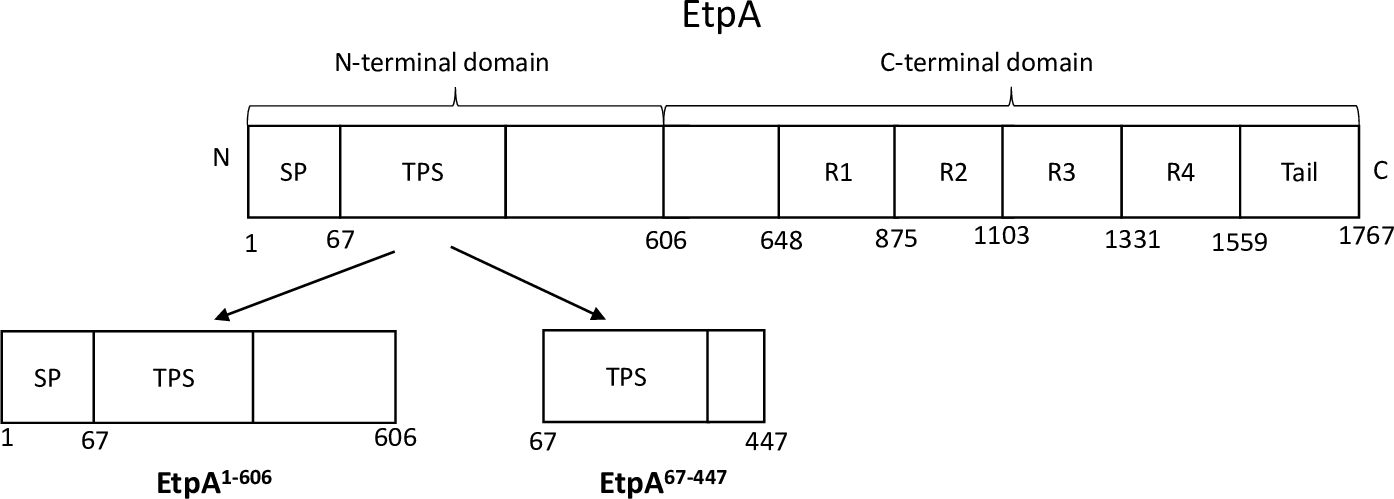

Supplement: S1 Fig — The signal peptide (SP) for localization and processing and the TPS domain for recognition by the transport partner are indicated. The four consecutive repeats (R1, R2, R3 and R4) and the C-terminal tail are also indicated. The numbers represent the start and end of each fragment. The two N-terminal fragments; EtpA1-606 and EtpA67-447 are shown. (TIF) [file pone.0287100.s002.tif]

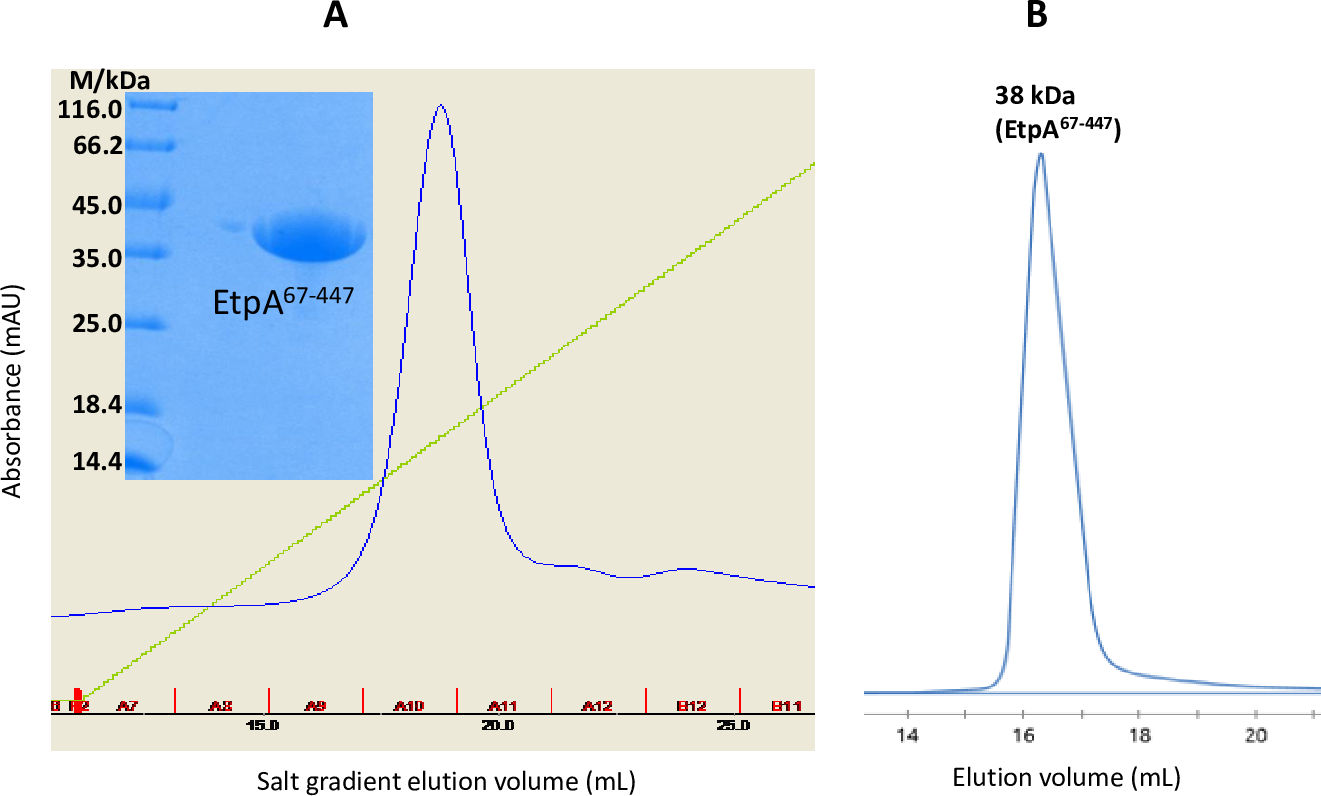

Supplement: S2 Fig — (A) Single-peak ion exchange chromatography profile, (B) Single-peak size exclusion chromatography profile of EtpA67-447 and a single band on SDS-PAGE (insert) matching the 38 kDa size of monomeric EtpA67-447. (TIF) [file pone.0287100.s003.tif]

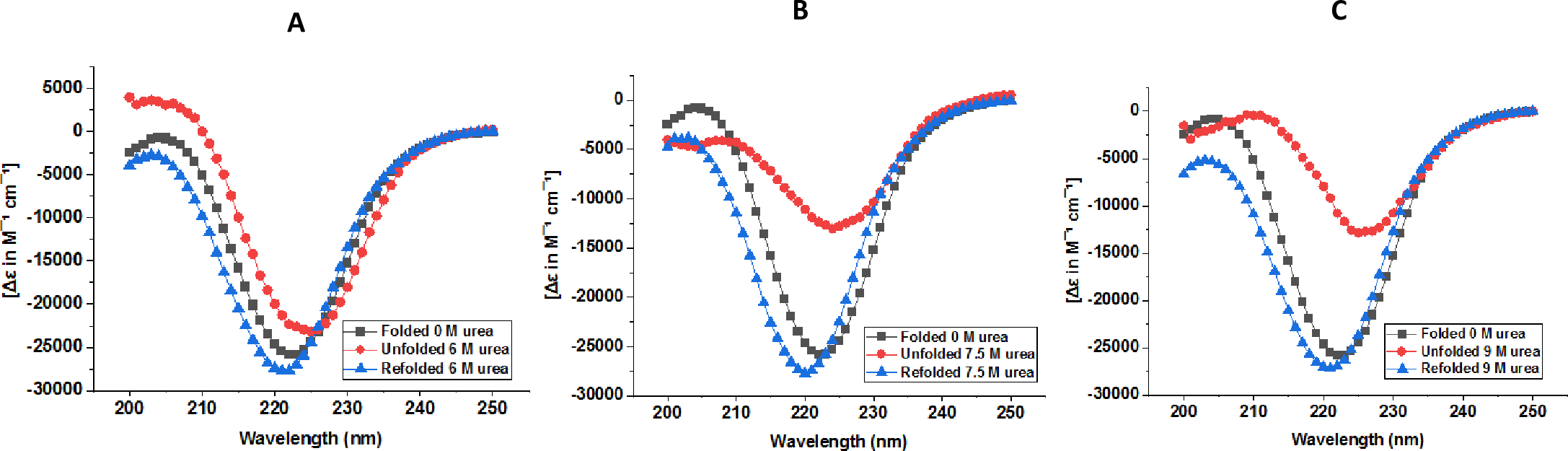

Supplement: S3 Fig — (A-C) CD spectra for untreated (0 M urea, black squares), urea treated (1.5 to 4.5 M urea, red spheres) and renatured samples (blue triangle). (TIF) [file pone.0287100.s004.tif]

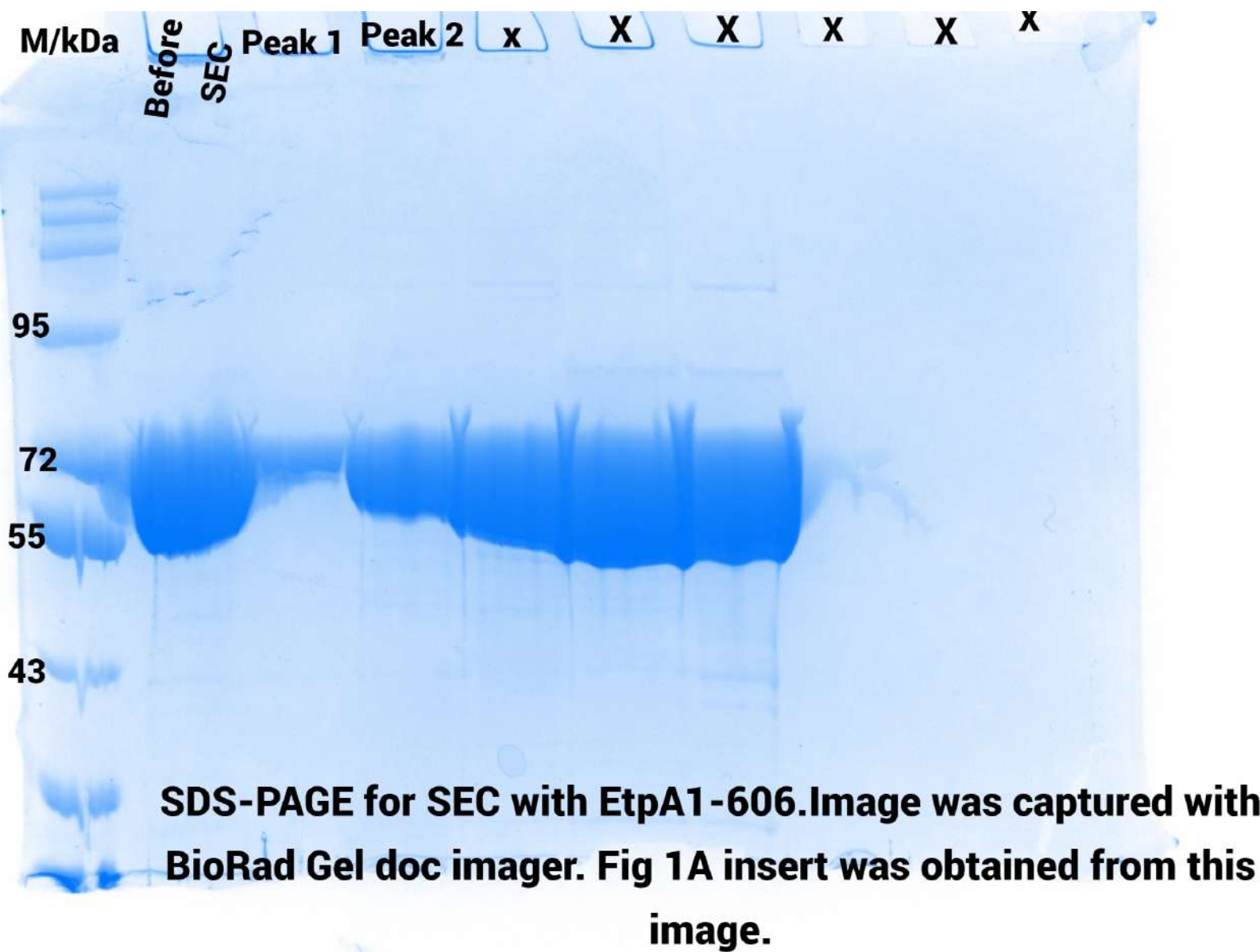

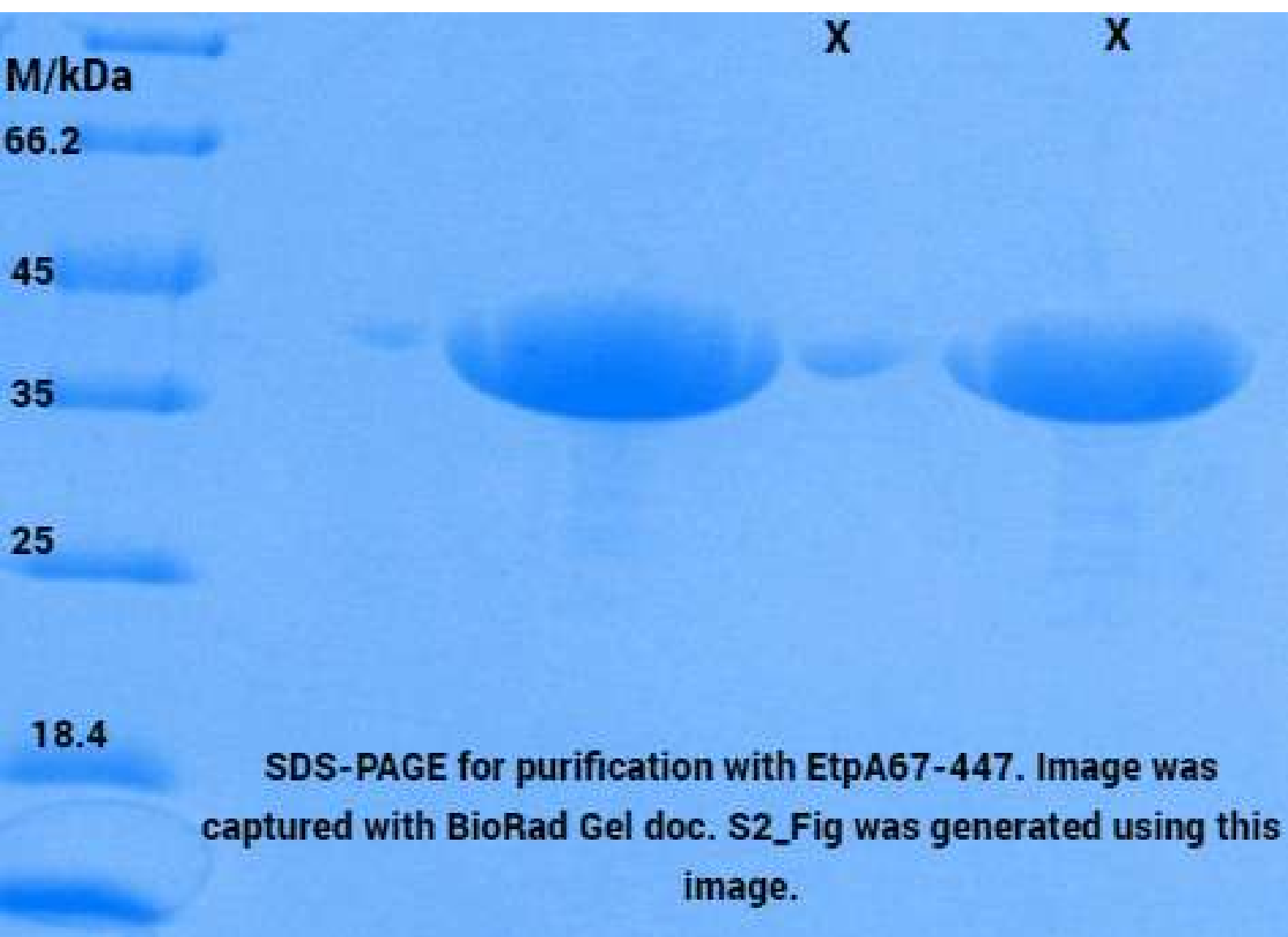

Supplement: S1 Raw images — (PDF) [file pone.0287100.s005.pdf]
